# Supplementary material for: Effect of stem cell therapies on tendon-bone healing after anterior cruciate ligament reconstruction in animal models: Protocol for a systematic review and meta-analysis
Source: PLoS One. 2026 Feb 4;21(2):e0341859. doi: 10.1371/journal.pone.0341859 (PMC12871951; doi:10.1371/journal.pone.0341859)
Supplement: S2 Table — (DOCX) [file pone.0341859.s002.docx]

Search strategies for the systematic review and meta-analysis on stem cell therapies for tendon–bone healing after anterior cruciate ligament (ACL) reconstruction in animal models. The following databases were searched from inception to August 2025.

| Database | Search strategy |
| --- | --- |
| PubMed | (("Anterior Cruciate Ligament"[Mesh] OR "anterior cruciate ligament"[tiab] OR ACL[tiab]) AND ("Anterior Cruciate Ligament Reconstruction"[tiab] OR reconstruct*[tiab] OR "Reconstructive Surgical Procedures"[Mesh] OR surgery[tiab] OR graft*[tiab]) AND ("Stem Cells"[Mesh] OR "Mesenchymal Stem Cells"[Mesh] OR "stem cell*"[tiab] OR "mesenchymal stem cell*"[tiab] OR MSC[tiab] OR "bone marrow stem cell*"[tiab] OR BMSC[tiab] OR "adipose-derived stem cell*"[tiab] OR ADSC[tiab] OR "tendon-derived stem cell*"[tiab] OR TDSC[tiab] OR "synovium-derived stem cell*"[tiab] OR "synovial stem cell*"[tiab] OR SDSC[tiab] OR "umbilical cord blood stem cell*"[tiab] OR "umbilical cord-derived mesenchymal stem cell*"[tiab] OR UCMSC[tiab] OR exosome*[tiab] OR "Extracellular Vesicles"[Mesh] OR "extracellular vesicle*"[tiab] OR EV[tiab] OR "Tissue Scaffolds"[Mesh] OR scaffold*[tiab]) AND (("Animals"[Mesh] NOT "Humans"[Mesh]) OR animal*[tiab] OR "in vivo"[tiab] OR preclinical[tiab] OR rat[tiab] OR rats[tiab] OR rabbit*[tiab] OR mouse[tiab] OR mice[tiab]) AND english[lang]) |
| Embase | (('anterior cruciate ligament'/exp OR 'anterior cruciate ligament':ti,ab OR acl:ti,ab) AND ('reconstruction'/exp OR 'reconstructive surgery'/exp OR reconstruct*:ti,ab OR surgery:ti,ab OR graft*:ti,ab) AND ('stem cell'/exp OR 'mesenchymal stem cell'/exp OR 'bone marrow stem cell'/exp OR 'adipose derived stem cell'/exp OR 'tendon derived stem cell'/exp OR 'synovium derived stem cell'/exp OR 'umbilical cord blood stem cell'/exp OR 'umbilical cord derived mesenchymal stem cell'/exp OR stem cell*:ti,ab OR mesenchymal stem cell*:ti,ab OR msc:ti,ab OR bmsc:ti,ab OR adsc:ti,ab OR tdsc:ti,ab OR sdsc:ti,ab OR ucmsc:ti,ab OR exosome*:ti,ab OR 'extracellular vesicle'/exp OR extracellular vesicle*:ti,ab OR ev:ti,ab OR 'tissue scaffold'/exp OR scaffold*:ti,ab) AND ('animal experiment'/exp OR 'in vivo study'/exp OR 'preclinical study'/exp OR animal*:ti,ab OR rat*:ti,ab OR rabbit*:ti,ab OR mouse:ti,ab OR mice:ti,ab) AND [english]/lim NOT ('human'/exp)) |
| Scopus | TITLE-ABS-KEY ( "anterior cruciate ligament" OR ACL ) AND TITLE-ABS-KEY ( reconstruct* OR "reconstructive surgery" OR surgery OR graft* ) AND TITLE-ABS-KEY ( "stem cell*" OR "mesenchymal stem cell*" OR MSC OR "bone marrow stem cell*" OR BMSC OR "adipose-derived stem cell*" OR ADSC OR "tendon-derived stem cell*" OR TDSC OR "synovium-derived stem cell*" OR "synovial stem cell*" OR SDSC OR "umbilical cord blood stem cell*" OR "umbilical cord-derived mesenchymal stem cell*" OR UCMSC OR exosome* OR "extracellular vesicle*" OR EV OR scaffold* ) AND TITLE-ABS-KEY ( animal* OR rat* OR rabbit* OR mouse OR mice OR preclinical OR "in vivo" ) AND ( LIMIT-TO ( LANGUAGE , "English" ) ) |
| SPORTDiscus | TX ( ("anterior cruciate ligament" OR ACL) ) AND TX ( (reconstruct* OR surgery OR graft*) ) AND TX ( ("stem cell*" OR "mesenchymal stem cell*" OR MSC OR "bone marrow stem cell*" OR BMSC OR "adipose-derived stem cell*" OR ADSC OR "tendon-derived stem cell*" OR TDSC OR "synovium-derived stem cell*" OR "synovial stem cell*" OR SDSC OR "umbilical cord blood stem cell*" OR "umbilical cord-derived mesenchymal stem cell*" OR UCMSC OR exosome* OR "extracellular vesicle*" OR EV OR scaffold*) ) AND TX ( animal* OR rat* OR rabbit* OR mouse OR mice OR preclinical OR "in vivo" ) |
| Cochrane Library | ("anterior cruciate ligament" OR ACL):ti,ab,kw AND (reconstruct* OR surgery OR graft*):ti,ab,kw AND ("stem cell*" OR "mesenchymal stem cell*" OR MSC OR "bone marrow stem cell*" OR BMSC OR "adipose-derived stem cell*" OR ADSC OR "tendon-derived stem cell*" OR TDSC OR "synovium-derived stem cell*" OR "synovial stem cell*" OR SDSC OR "umbilical cord blood stem cell*" OR "umbilical cord-derived mesenchymal stem cell*" OR UCMSC OR exosome* OR "extracellular vesicle*" OR EV OR scaffold*):ti,ab,kw AND (animal* OR rat* OR rabbit* OR mouse OR mice OR preclinical OR "in vivo"):ti,ab,kw |
| Web of Science | TS=(("anterior cruciate ligament" OR ACL) AND (reconstruct* OR surgery OR graft*) AND ("stem cell*" OR "mesenchymal stem cell*" OR MSC OR "bone marrow stem cell*" OR BMSC OR "adipose-derived stem cell*" OR ADSC OR "tendon-derived stem cell*" OR TDSC OR "synovium-derived stem cell*" OR "synovial stem cell*" OR SDSC OR "umbilical cord blood stem cell*" OR "umbilical cord-derived mesenchymal stem cell*" OR UCMSC OR exosome* OR "extracellular vesicle*" OR EV OR scaffold*) AND (animal* OR rat* OR rabbit* OR mouse OR mice OR preclinical OR "in vivo")) |
